# Decision-making capacity regarding healthcare, welfare and finances in a secure forensic setting

**DOI:** 10.1192/j.eurpsy.2022.888

**Published:** 2022-09-01

**Authors:** K. Tong, C. Har, H. Kennedy, M. Davoren

**Affiliations:** 1 National Forensic Mental Health Service, Central Mental Hospital, Dundrum, Dublin, Ireland; 2 Central Mental Hospital and Trinity College Dublin, Dundrum Centre For Forensic Excellence, Dublin, Ireland

**Keywords:** mental illness, functional capacity, decision-making capacity, length of hospital stay

## Abstract

**Introduction:**

Impairment in decision-making capacity is a serious consequence of executive dysfunction secondary to serious mental disorders like schizophrenia. Functional mental capacity (FMC) refers to an individual’s ability to make and communicate legally competent decisions autonomously. Studies have shown that FMC is dependent on severity of psychosis and can improve with treatment.

**Objectives:**

To ascertain the correlation between the scores on a structured judgement tool, namely the Dundrum Capacity Ladders (DCL) with level of acuity of treatment setting and length of stay in a secure forensic hospital.

**Methods:**

Sixty-two patients were interviewed using the DCL across three domains – healthcare, welfare and finances. Correlation between DCL scores, length of hospital stay and level of acuity of treatment setting was assessed.

**Results:**

As patients moved from higher to lower dependency wards, mean DCL score increased, indicating a higher level of capacity. Patients in high dependency wards were most impaired while those in the low dependency wards performed significantly better (r_s_=0.472, p<0.001). The longer the patients stayed in the hospital, up until five years, the higher the mean welfare domain score (r_s_=0.402, p=0.011) and mean DCL score (r_s_=0.376, p=0.018). Beyond five years of hospital stay, those who had lower DCL scores and did not improve had longer length of stay.

**Conclusions:**

Patients’ FMC improve as they progress from high to low level of acuity of treatment setting. However, this is dependent on the length of hospital stay. FMC may be a measure of recovery in the forensic setting.

**Disclosure:**

No significant relationships.

